# Eclampsia and Its Treatment Modalities: A Review Article

**DOI:** 10.7759/cureus.29080

**Published:** 2022-09-12

**Authors:** Shivani Akre, Kapil Sharma, Swarupa Chakole, Mayur B Wanjari

**Affiliations:** 1 Department of Medicine, Jawaharlal Nehru Medical College, Datta Meghe Institute of Medical Sciences, Wardha, IND; 2 Research, Jawaharlal Nehru Medical College, Datta Meghe Institute of Medical Sciences, Wardha, IND

**Keywords:** hypertension, end organ dysfunction, proteinuria, seizures, eclampsia

## Abstract

Hypertension in pregnancy is one of the major contributors to mortality and morbidity. Pregnant women and fetuses are both at high risk of the severe complications of preeclampsia known as eclampsia. Eclampsia is a disorder that requires immediate detection and treatment. Eclampsia and preeclampsia during pregnancy are known to cause morbidity and even death in both the mother and fetus if not properly diagnosed. Chronic hypertension, prenatal hypertension, preeclampsia on top of chronic hypertension, and eclampsia are the four types of hypertension. Preeclampsia is the precursor to eclampsia. Associated with end-organ failure and proteinuria after 20 weeks of pregnancy, preeclampsia is characterized by the development of hypertension with systolic blood pressure (BP) of at least 140 mmHg and/or diastolic BP of at least 90 mmHg. It can lead to the failure of the liver, thrombocytopenia, pulmonary edema, central nervous system (CNS) abnormalities, and renal dysfunction. The emergence of new generalized tonic-clonic seizures in a pregnant woman with preeclampsia is known as eclampsia. Eclamptic seizures can happen prior to delivery, 20 weeks following conception, during delivery, and after delivery. Although rare, gestational trophoblastic illness has been associated with seizures that start before 20 weeks. In this article, we examine the pathogenesis, causes, signs, symptoms, and treatment modalities in patients with eclampsia.

## Introduction and background

Earlier, eclampsia was perceived as a condition that culminated in delivery and was marked by convulsive convulsions typical of late pregnancy. The edema of Bright's illness, a sudden glomerulonephritis start marked by proteinuria, and the bloated look of women who had convulsions were both noted by scientists from the late 19th century, who were firm believers in scientific empiricism. It is important for a physician to look for signs and symptoms of eclampsia as it can help prevent and decrease mortality in many cases, especially primigravida patients, who are generally more prone to develop pregnancy-induced hypertension (PIH) after 20 weeks of pregnancy. Patients may go into a seizure attack even after delivery, and hence it is advised to give MgSO4 even after delivery as a maintenance dosage [1-2]. A clinician should always look for side effects of MgSO4 as it can lead to respiratory arrest and can cause mortality in patients. The best treatment for eclampsia is to abort or deliver the fetus, whichever is possible according to the age of gestation. Proteinuria in childbearing women with seizures was discovered following an investigation into urine changes in them. Also, increased blood pressure (BP) was also observed in these women. Thus, it was defined as eclampsia, and it came to be understood that these events could lead to a series of consequences for maternal and fetal lives. Even though preeclampsia has been better understood, the specific etiology of eclampsia is still unknown. It is hypothesized that preeclampsia results in increased blood-brain barrier permeability, which alters cerebral blood flow due to decreased autoregulation. The majority of women who experience numerous seizures due to eclampsia show signs of cerebral infarction and HELLP syndrome, making the avoidance of their occurrence crucial. Pulmonary edema, postpartum hemorrhage, disseminated intravascular coagulation, and acute renal failure are some of the other complications of eclampsia [3]. Also, it can cause intrauterine growth restriction in the fetus.

## Review

Pathogenesis and causative risk factors

The primary pathophysiology in eclampsia responsible for the central nervous system (CNS)-related signs and symptoms is severe diffuse cerebral vasospasm that results in deficits in cerebral perfusion and cerebral edema. However, in preeclampsia, the vascular remodeling in placental tissue is absent, which causes the maternal spiral arterioles to undergo intense vasospasm, by first producing capillary leak, then systemic vascular dysfunction, and finally vasospasm. This also results in proteinuria, glomerular endotheliosis, HELLP syndrome-like coagulation abnormalities, cerebral edema (eclampsia), elevated angiotensin-II sensitivity, and hypertension. Oxidative stress results in endothelial damage and the production of free radicals and cytokines, including vascular endothelial growth factor I. Additionally, maternal endothelium function is severely impacted by angiogenic or pro-inflammatory proteins. Endothelial disturbance affects the cerebral endothelium as well as the uterine one, resulting in neurological illnesses such as eclampsia [2].

**Figure 1:**
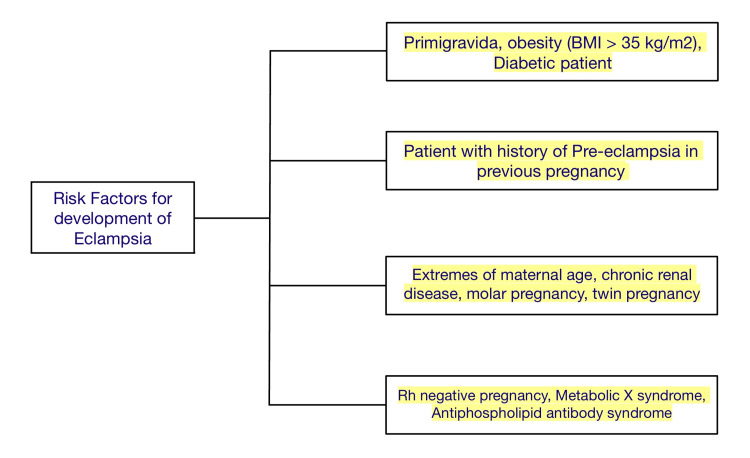
Risk factors for eclampsia development in a pregnant female patient

The risk factors of preeclampsia and eclampsia are chronic hypertension, diabetes, chronic renal failure, systemic lupus erythematosus, vascular diseases, and antiphospholipid antibody syndrome (APLA). A previous history of preeclampsia increases the chances of the same in a subsequent pregnancy by 30%. Other risk factors are multifetal pregnancy, extremes of age, molar pregnancy, obesity, assisted reproductive techniques, and intrauterine growth restriction (IUGR)/abruption/intrauterine death (IUD) in previous pregnancies. Smoking is a protective factor against preeclampsia while the placenta previa has a negative association with it (Figure 1).

Prevention of preeclampsia and eclampsia

According to the observational research design, there is a significant link between vitamin D insufficiency and preeclampsia incidence, as well as a risk of preeclampsia when blood 25(OH)D (25-hydroxyvitamin D) concentrations are below 50 nmol/L (20 ng/mL). However, when the shortfall was specified as 38 nmol/L (15.2 ng/mL), the association was not apparent. The method may not be evident, but BP, electrolyte balance, and plasma volume homeostasis are all regulated by the renin-angiotensin system, a regulatory cascade. Therefore, by inhibiting the renin-angiotensin system, normal blood vitamin D levels help prevent hypertension (Table 1) [4].

**Table 1:** Steps for preventing preeclamptic and eclamptic episodes

Sr. no.	Steps
1	Eating no added salt or using little salt in meals
2	Drinking plenty of water per day per hour and going for frequent urination after drinking water hourly also helps in preventing urinary tract infections
3	Avoiding fatty and junk foods, avoiding fried oily food items
4	Getting enough rest
5	Exercising regularly and going for a 30-minute walk also helps; advise the patient to elevate her feet several times during the day
6	It is best to refrain from consuming alcohol and caffeinated beverages
7	Taking medications regularly on time as prescribed by your obstetrician is very important

Signs and symptoms

The signs and symptoms of eclampsia include seizures, extreme agitation, and unconsciousness. The majority of women experience the following preeclampsia symptoms before the seizure: nausea and vomiting, stomachaches, epigastric discomfort, headaches (due to stretching of the liver capsule), swelling of the hands and face, and difficulties with eyesight including loss of vision, double vision, blurry vision, and missing portions of the visual field [5].

Signs of eclampsia include BP of more than 160/110 mmHg, platelet count of less than one lakh, serum creatinine of more than 1.1 mg/dl, liver transaminases at least two times over the average, pulmonary edema, new onset of headache that does not respond to usual analgesics, and visual symptoms like blurring of vision, flashes of light, and scotomas. Severe preeclampsia with unexpected headache onset or epigastric pain or visual symptoms is called impending eclampsia.

Clinical Case Example (Own Patient Case)

A 22-year-old primigravida was examined for a normal checkup at the outpatient prenatal clinic. She was 32 weeks pregnant, according to the first-trimester ultrasound. For the previous 24 hours, she had been suffering from a strong, ongoing mid-epigastric discomfort and a headache behind the ear that was not eased by acetaminophen, as well as visual spots and flashes of light. She had gained 10 pounds since her previous appointment two weeks earlier. Upon assessment, her BP was 165/115 mmHg. Her fingers seemed swollen, and she had two or more pedal edema. The fundal height was 29 cm, and regular fetal heartbeats were 145 beats per minute. A urine spot dipstick revealed 4+ protein.

HELLP syndrome

HELLP syndrome is one of the fatal consequences of eclampsia. Diagnosis of HELLP syndrome is made based on the presence of an obstetric triad of (1) serum bilirubin over 1.2 mg/dl, presence of schistocytes on peripheral smear, low serum haptoglobin or increased LDH, and severe anemia unrelated to blood loss are required for the diagnosis of hemolysis; (2) increased liver enzyme levels; and (3) less than 100,000 platelets in the blood.

Clinical Case Example (Own Patient Case)

A 30-year-old multigravida presented with a 32-week pregnancy. Her BP was measured during a usual pregnancy checkup and found to be 160/105 mmHg. Prior BP results had been normal. A preeclampsia workup was started, and the results showed high levels, including a platelet count of 85,000, total bilirubin, lactate dehydrogenase, alanine aminotransferase, and aspartate aminotransferase. She denied experiencing headaches or changes in her vision. This condition most commonly presents in the third trimester, and the most common presentation involves pain epigastrium or right upper quadrant, more common in multiparous; in up to 15% of cases, the BP may be normal, and differential diagnosis is acute fatty liver of pregnancy, which is a more severe form of liver injury and presents as liver failure.

Treatment

The treatment involves administering prophylactic MgSO4. Severe hypertension should be treated with anti-hypertensive agents like labetalol, hydralazine, alpha-methyldopa, nifedipine (CBB), and sodium nitroprusside (last-resort drug). For pregnancies of more than or equal to 34 weeks, gestation delivery should be done after maternal stabilization. For pregnancies of less than 34 weeks where maternal and fetal status is reassuring, delivery after a course of steroids for fetal lung maturation should be considered [6-9]. HELLP syndrome is diagnosed with the Tennessee criteria. It is managed by immediate termination of pregnancy by induction of labor (IOL).

Management of patients with preeclampsia

Severe preeclampsia symptoms at any stage of pregnancy and signs of maternal or fetal risk call for an aggressive, quick delivery. Preventing seizures and managing BP are the key objectives. IV MgSO4 4 gm and 10 gm IM as loading dose based on Pritchard's regimen should be administered followed by 5 gm every four hours until 24 hours after delivery. BP should be reduced using IV labetalol and/or hydralazine to diastolic levels of 90-100 mm Hg if BP ≥160/110 oral else oral labetalol should be used. When there is no risk to the mother or the fetus and the gestational age is between 26 and 34 weeks, conservative inpatient therapy may occasionally be tried if BP can be brought down to below 160/110 mmHg. It is recommended to provide maternal betamethasone to promote fetal lung maturation. Termination of pregnancy is the definitive management of severe preeclampsia at or more than 34 weeks of gestation [10-13].

Management of patients with eclampsia

The mother's airway and tongue must first be shielded. IV MgSO4 4 gm and 10 gm IM as loading dose based on Pritchard's regimen should be administered followed by 5 gm every four hours until 24 hours after delivery. BP should be reduced to diastolic levels of 90-100 mmHg with IV labetalol and/or hydralazine if BP ≥160/110. For eclampsia at any gestational age, aggressive early delivery is advised when the mother and fetus have stabilized. If the mother and fetus are stable, vaginal birth with an IV oxytocin infusion should be tried [14-16].

MgSO4

MgSO4 is the drug of choice for the prevention of seizures in severe preeclampsia as well as in the prevention of the recurrence of seizures in eclampsia. It raises the seizure threshold by acting on the NMDA receptor and also causes cerebral vasodilatation. It is not an anti-hypertensive agent. It undergoes renal excretion. Its loading dose does not change with renal insufficiency; only the maintenance dose changes. The maintenance dose is to be given if the patellar reflex is present, respiratory rate is >12/minute, urine output exceeds 100 ml in four hours, and therapeutic blood levels are 4-7 meQ/L. The first sign of toxicity is the loss of patellar reflex. The antidote for its toxicity is 10 ml of 10% IV calcium gluconate [17].

PIH is more common in primigravida females. It is associated with the large size of the placenta like in cases of twin pregnancies, diabetes, RH-negative, and molar pregnancy. PIH is more common in patients with polycystic ovarian syndrome (PCOS), antiphospholipid antibody syndrome (APLA), and metabolic X syndrome. In a molar pregnancy, PIH happens before 20 weeks of pregnancy. The most common mode of delivery in PIH is vaginal. During vaginal delivery, epidural analgesia may be given for the pain. Patients should be catheterized. The second stage of labor should be cut short by the prophylactic use of forceps/vacuum. The use of methylergometrine is contraindicated as it raises blood pressure. The indications for cesarean section (C-section) in PIH are as follows: poor BISHOP score [18], absent end-diastolic blood flow on the Doppler scan, and reversed end-diastolic blood flow on the doppler scan. The anesthesia of choice in a patient with PIH is epidural (spinal anesthesia is given normally). If there is an absent or reverse end-diastolic flow, general anesthesia is given.

## Conclusions

Globally, preeclampsia is the main cause of maternal morbidity and mortality. Preeclampsia's postpartum and long-term consequences have grown in number and importance, despite the fact that preeclampsia-eclampsia mortality has dropped dramatically in the United States due to enhanced perinatal surveillance and early therapies. We now understand that women with preeclampsia are more prone to dementia and cardiovascular diseases (CVD) later in life, including immediately lethal heart attack without the gradual, warning signs of acute coronary syndrome. It is unclear if the risk precedes and muddles preeclampsia or is a result of it. To reduce the danger of CVD and its possibly deadly side effects, interventional studies must get more attention during the postpartum, asymptomatic period.
